# Bilingualism is a long-term cognitively challenging experience that modulates metabolite concentrations in the healthy brain

**DOI:** 10.1038/s41598-021-86443-4

**Published:** 2021-03-29

**Authors:** Christos Pliatsikas, S. M. Pereira Soares, T. Voits, V. Deluca, J. Rothman

**Affiliations:** 1 School of Psychology and Clinical Language Sciences, University of Reading, Harry Pitt Building, Earley Gate, Whiteknights Road, Reading, RG6 6AL UK; 2 Centro de Ciencia Cognitiva, Facultad de Lenguas y Educación, Universidad Antonio de Nebrija, Calle de Sta. Cruz de Marcenado, 27, 28015 Madrid, Spain; 3 Department of Linguistics, University of Konstanz, Universitätsstraße 10, 78464 Konstanz, Germany; 4 Department of Language and Culture, The University of Tromsø, Hansine Hansens veg 18, 9019 Tromsø, Norway

**Keywords:** Language, Cellular neuroscience

## Abstract

Cognitively demanding experiences, including complex skill acquisition and processing, have been shown to induce brain adaptations, at least at the macroscopic level, e.g. on brain volume and/or functional connectivity. However, the neurobiological bases of these adaptations, including at the cellular level, are unclear and understudied. Here we use bilingualism as a case study to investigate the metabolic correlates of experience-based brain adaptations. We employ Magnetic Resonance Spectroscopy to measure metabolite concentrations in the basal ganglia, a region critical to language control which is reshaped by bilingualism. Our results show increased myo-Inositol and decreased *N*-acetyl aspartate concentrations in bilinguals compared to monolinguals. Both metabolites are linked to synaptic pruning, a process underlying experience-based brain restructuring. Interestingly, both concentrations correlate with relative amount of bilingual engagement. This suggests that degree of long-term cognitive experiences matters at the level of metabolic concentrations, which might accompany, if not drive, macroscopic brain adaptations.

## Introduction

Research over the past two decades has unequivocally shown that human brain structure is not static. Rather, it is affected by learning new skills via environmental experiences of individuals. Notably, this malleability applies to the healthy brain as well as to the diseased one, and appears to be relatively independent of critical periods and other developmental milestones^1,2^. For example, grey and white matter adaptations have been reported for cognitively demanding experiences such as learning a new language, mastering complex visuospatial tasks and memory training^3–5^. Crucially, these adaptations tend to be observed in brain regions significantly associated with the task at hand, as well as white matter tracts that provide connectivity between implicated regions. Moreover, the adaptations themselves are not static but dynamic, and dependent on the quality and quantity of the experiences that induce them, e.g. the intensity and degree of novel skill training^4^ and bilingualism^6^.

Although the exact processes underlying observed structural neuroplasticity are still under investigation, several suggestions link them to cognitive experiences^2,4^. For example, volumetric changes in grey matter structure have been interpreted as a mechanism that temporarily increases the availability of neural pathways for a new skill to be acquired, most likely via the generation of new dendritic spines. This is subsequently followed by pruning of idle connections and spines while retaining the most efficient networks. In turn, this leads to volumetric renormalisation of previously grown regions^4^. Conversely, changes to white matter structure have been interpreted as increases in the availability of myelin as a result of increased axonal activity, which, in turn, can be brought about by new skill learning. These effects are very likely to be linked to increased activity of glial cells, especially oligodendrocytes^2^.

Most of the relevant evidence in humans comes from MRI studies that utilise macroscopic indices, such as volume and thickness for grey matter, fractional anisotropy and mean diffusivity for white matter. However, it is worth noting that all proposed processes are energy demanding. Therefore, there is scope for studying neuroplasticity at the level of brain metabolism, specifically with methods that tap into local metabolic activity and the neurochemical processes that underlie it. A method that has been increasingly used for this purpose is proton magnetic resonance spectroscopy (^1^H-MRS)^7^. MRS has typically been used in clinical settings in order to estimate average metabolite concentrations in a specific region of interest (RoI) in the brain in vivo^8^. To date, MRS research has mainly focused on a few major metabolites. These include: *N*-acetylaspartate (NAA), a marker of neural density and viability, Choline (CHO), related to the density and integrity of the cell membrane, Creatine (CRE), considered essential in cellular energy metabolism, Glutamate + Glutamine (GLX), which play a role in detoxification and regulation of neurotransmitters, and myo-Inositol (INS), a marker of glial proliferation and glial size (for review, see^7^).

Disruptions of metabolic activity can relate to a breakdown of cognitive functions; as such, metabolic activity has been studied in neurodegenerative diseases such as Parkinson’s^9^, Huntington’s^10^ and Alzheimer’s^11^ Diseases, Multiple Sclerosis^12^, and Primary Progressive Aphasia^13^, where disruptions have been typically treated as precursors of disease in pre-symptomatic patients, and/or as predictors for related cognitive deficits. Crucially, similar approaches have been used with healthy participants, where metabolite concentrations have been used as predictors of cognitive functionality in domains such as reading^14^, episodic memory^15^, and executive control^16^ among others. In the domain of ageing, while metabolite concentrations are also typically used as predictors of cognitive abilities, it is not uncommon that they are also treated as *outcomes* of the ageing processes (for a review, see^17^). For example, Chiu et al.^18^ suggest that changing levels of metabolites such as CHO, CRE and NAA in the elderly can be viewed as a proxy for the study of several age-related neural processes. These include glial proliferation, which acts as a compensatory mechanism in challenging situations where there is an increased energy demand that cannot be supported by regional blood flow. It is worth noting that these changes can vary between different metabolites and different brain regions; specifically, NAA has been shown to *decrease* in the basal ganglia but *increase* in the ACC, INS has generally been shown to increase with age, and CHO and CRE to stay stable, whereas GLX is less well studied and understood, especially as far as subcortical regions are concerned (for a recent systematic review, see^19^).

In light of the above, it is reasonable to investigate whether, and to what extent, different types of cognitively demanding lifestyle enrichment activities might induce comparable changes to metabolite concentrations, even at younger ages. Recall that skill acquisition and maintenance entail increased cognitive demands and have been shown to bring about structural brain plasticity and accompanying increased energy demands. In turn, it could follow that skill acquisition and maintenance would be accompanied by metabolic changes, similar to what has been found for aging. Indeed, and related to the present investigation, recent MRS research has suggested that changes in metabolite concentrations are potentially good markers of experience-related structural brain plasticity, potentially signifying neuronal, glial and vascular changes as responses to cognitively challenging tasks^20^. For example, several studies have reported increased levels of INS, CHO and CRE in the occipital cortex of blind subjects, compared to sighted ones^21,22^, and these findings have been interpreted as markers of plastic changes in the glial cells (astrocytes and oligodendrocytes) that underlie and support repurposing of the visual cortical regions for other sensory modalities in the blind. This suggestion corroborates previous findings of functional and structural adaptations of the visual cortex in the blind (for reviews, see^23,24^), suggesting that changes at the macroscopic level are very likely accompanied by metabolic effects. Importantly, changes in the concentrations of INS, NAA and GLX have also been reported in the occipital cortex of sighted participants after visual training^25^. This suggests that the glial cells might have a central role in brain restructuring as a result of cognitive and sensory experiences, beyond ageing or pathology.

In line with the above, it is reasonable to hypothesise and seek to empirically test the extent to which similar effects in metabolite concentrations can result from long-term, cognitively challenging lifestyle enrichment factors known to affect brain structure and function. One of these dynamic factors is bilingualism. It is widely accepted that the mental juggling of more than one language in a single mind/brain is cognitively demanding^26^. Indeed, the need for use between the two languages is unpredictable. As a result, both languages are continuously active at all times irrespective of apparent need or intent. This reality requires an efficient system of control for appropriate selection of one language for comprehension and production alongside simultaneous suppression of the irrelevant language to a low level of idle activation for whenever the other may become needed^27,28^. This constant competition taxes domain general executive control and its underlying brain structures, leading to long-term adaptations in domain general cognition^29^, and in brain function^30^, structure^6^ and metabolism^31^. Notably, such effects can be both observed after extended long-term exposure in a bilingual environment^32,33^ and after short-term intensive language training^34,35^. Moreover, recent literature has shown that the nature and location of these effects is modulated by quantitative measures of the depth and intensity of engagement bilinguals have with using their languages in diverse contexts and, thus, opportunities to switch between them^36–39^.

Overall, it seems reasonable to predict that the continuously challenging task of handling two or even more languages would result in brain changes at the neurochemical level. After all, bilingualism requires greater and more sustained efficiency in brain regions subserving language and cognitive control, such as the anterior cingulate gyrus (ACC) and parts of the basal ganglia such as the caudate nucleus and putamen. In fact, all these regions have been shown to change in shape and/or volume as a response to bilingualism^27,40–43^. Therefore, it is possible that these structural changes might have their correlates in changes in metabolite concentrations. For example, and drawing parallels from the findings from healthy ageing and neuroplasticity in the blind^21,22^, the observed restructuring of these regions might be characterised, at least partly, by expansion of glial cells, which is in itself marked by increases in regional CHO, CRE and INS. Crucially, this hypothesis could provide the biological basis of bilingualism-induced regional neuroplasticity, which is currently not well understood^6^.

To the best of our knowledge, only one study has looked at correlates in metabolic concentrations of the effects of bilingualism on cognition and the brain. Weekes et al.^44^ compared young bilingual adults and age- and education-matched monolingual controls on metabolite concentrations (NAA, CHO, CRE, INS and GLX) in the anterior cingulate cortex (ACC), a region critical for domain general cognitive control^27^. Contrary to their predictions, Weekes and colleagues reported *lower* levels of absolute NAA, CHO, CRE and GLX concentrations in bilinguals than monolinguals, and interpreted this finding as an indication of more efficient control monitoring of the bilingual brain as a result of prolonged bilingual experience. However, they reported no significant correlations between these concentrations and executive control abilities as measured by a Flanker Task, nor a significant difference in task performance between the two groups. This study suggests that there might be effects of bilingualism on brain metabolism that are not detectable behaviourally, echoing some evidence in the neuroimaging literature suggesting that behavioural measures might not always capture latent effects of bilingualism on brain function^37^. Apropos the present study, Weekes et al.^44^ set of results provided the first evidence that bilingualism-induced neuroplasticity might have its roots in changes in metabolite concentrations.

The present study expands on Weekes et al.^44^ by looking at the effects of bilingualism on metabolite concentrations in the basal ganglia, specifically the caudate nucleus and the putamen. These are key structures for language selection and cognitive control^27^ shown to be affected structurally by bilingualism^40,42^. In order to study such effects across the adult lifespan, the present study comprises a relatively large sample that spans an age range representative of the adult lifespan, additionally accounting for the known effects of age on metabolite concentrations^17^. Similar to Weekes et al.^44^, we looked at absolute concentrations of five key metabolites (NAA, INS, CHO, GLX and CRE). Based on previous literature, we predicted that increased age will lead to increased INS and decreased NAA concentrations in the basal ganglia, potentially accompanied by increases in GLX but not CHO or CRE^17–19^. In terms of the effects of bilingualism, we consider the data in two ways: following the most traditional practice in the field^44^, we split our participant groups into Bilinguals and Monolinguals according to experiences in using more than one language (for details, see “Methods”). For this comparison, we predicted overall higher concentrations of INS and CHO in bilinguals, compared to monolinguals, an effect that would signify glial expansion, which could contribute to the observed restructuring of the basal ganglia in bilinguals^21,22^. Moreover, if the effects of age and bilingualism are based on similar mechanisms, then the combined effect of these two factors should lead to steeper increases with age of the INS and CHO concentrations in bilinguals. Following from more recent suggestions regarding the determinant role that engagement with bilingual experiences at the individual level has in adapting brain structure and function differentially^37,38^, we disposed of the monolingual versus bilingual comparison and considered our entire sample to investigate whether metabolite concentrations of interest are modulated by intensity of engagement. We predicted that the more intense and sustained the bilingual experience, the greater the concentration changes would manifest.

## Results

### Effects of age and bilingualism

**Figure 1:**
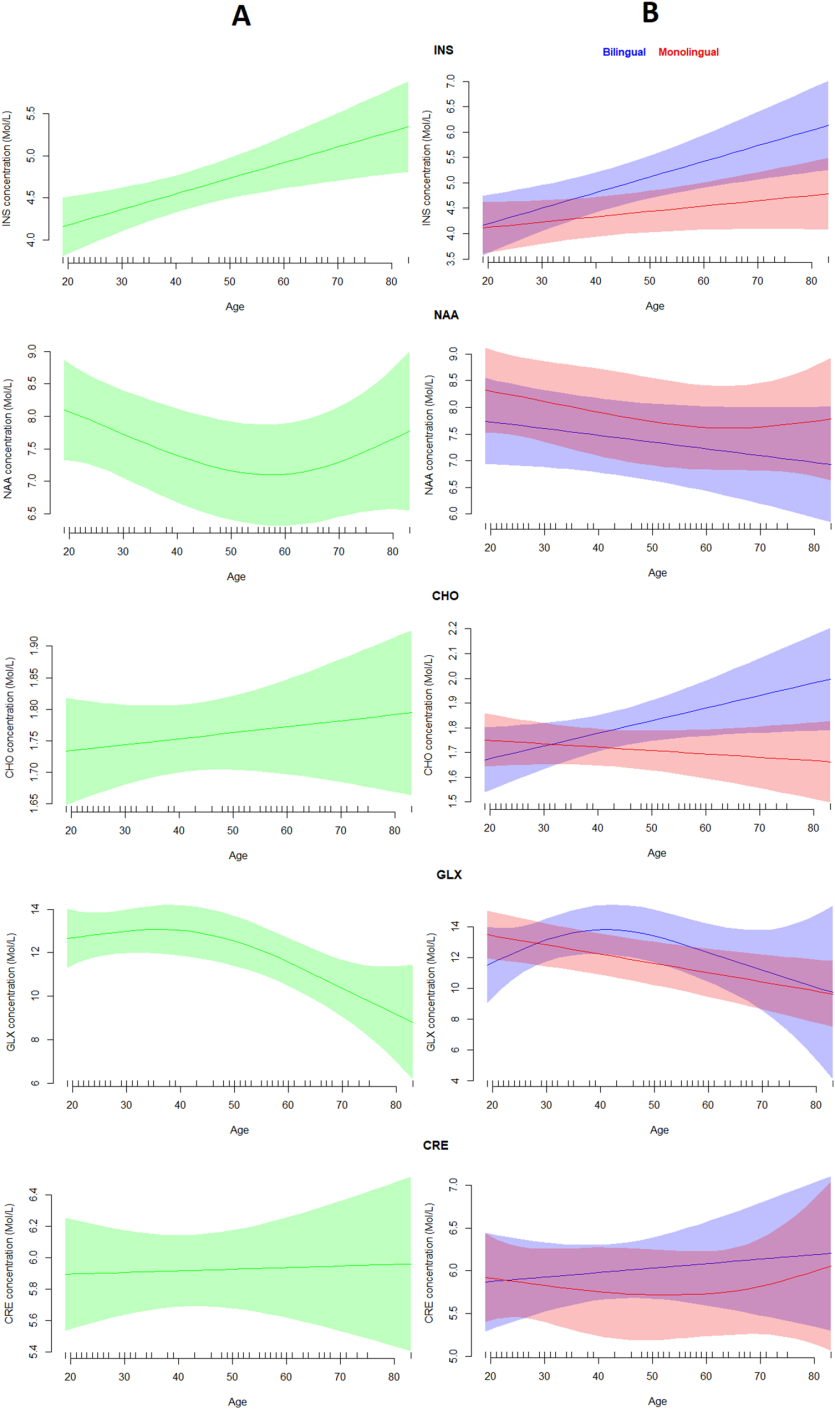
(**A**) The effects of Age on the concentrations of the metabolites of interest; (**B**) the effects of Age split by group. Shaded regions represent 95% confidence bands for the smoothed effects. Image produced in R ver. 4.0.3 (https://www.r-project.org/).

The results from the first set of analyses are illustrated in Table 1. The analysis revealed that Age was a significant predictor of the concentrations of NAA, INS and GLX only. Specifically, while INS concentration significantly increased with age, NAA and GLX concentrations significantly decreased. Moreover, Bilinguals were found to have a significantly higher concentration of INS and lower concentration of NAA than Monolinguals. There was also a significant Age x Bilingualism interaction for CHO only, suggesting that age was a significant predictor of CHO concentrations for bilinguals (p = 0.038) but not for monolinguals (p = 0.445). Figure 1 illustrates the concentrations per group across age for each metabolite.

**Table 1:** The effects of Age and Bilingualism on the absolute metabolite concentrations, expressed in p values.

	INS	NAA	CHO	GLX	CRE
Age	0.003*	0.012*^	0.519	0.005*^	0.870
Bilingualism	0.020*	0.050*	0.212	0.608	0.535
Age × bilingualism	0.101	0.847	0.035*	0.190	0.615

For significant effects all Fs > 3.3

^edf > 1, denoting a non-linear effect.

### Effects of individual bilingual experiences

**Figure 2:**
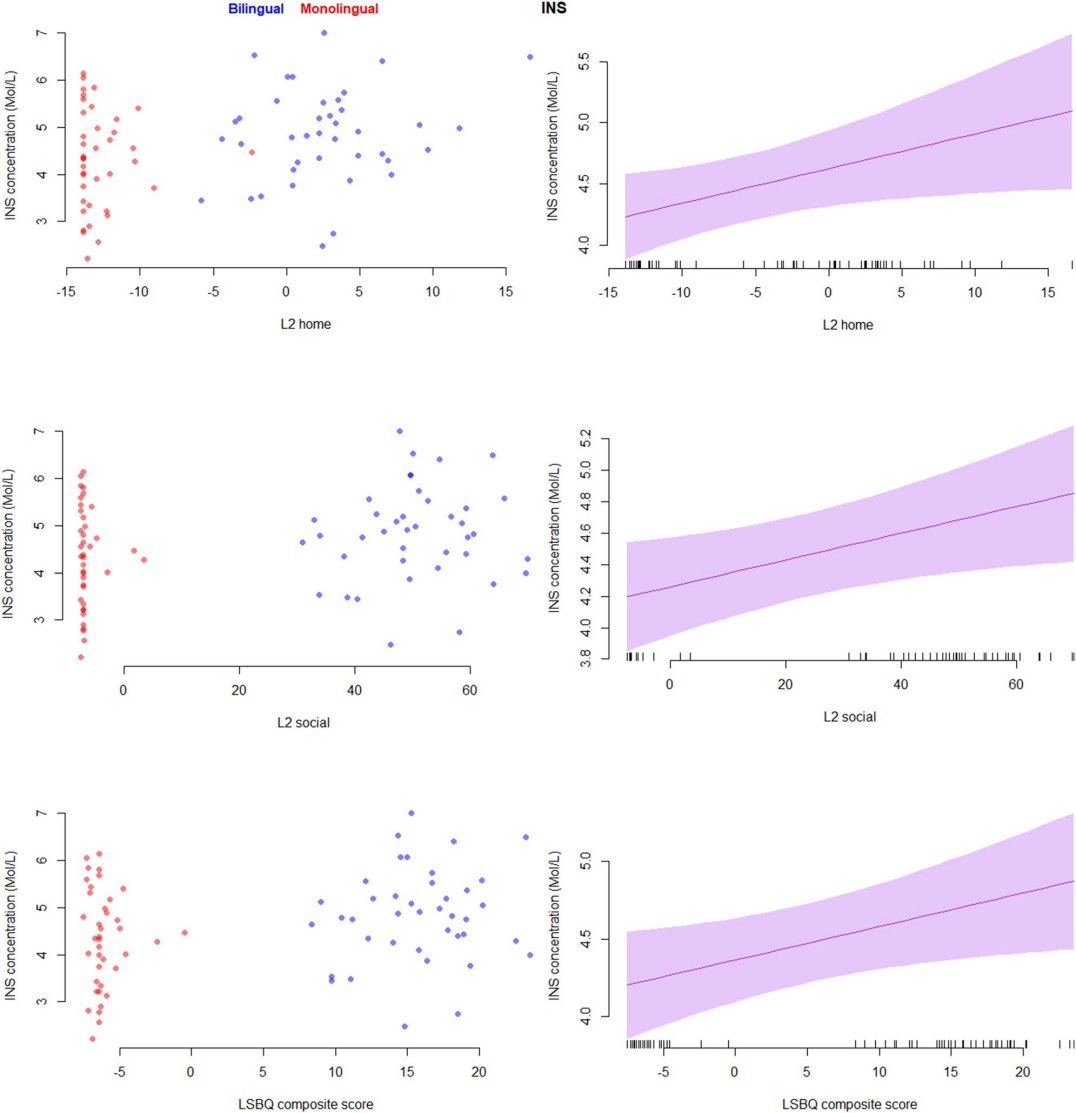
L2 home, L2 social and LSBQ composite scores as predictors of the absolute INS concentrations. Shaded regions represent 95% confidence bands for the smoothed effects. Image produced in R ver. 4.0.3 (https://www.r-project.org/).

**Figure 3:**
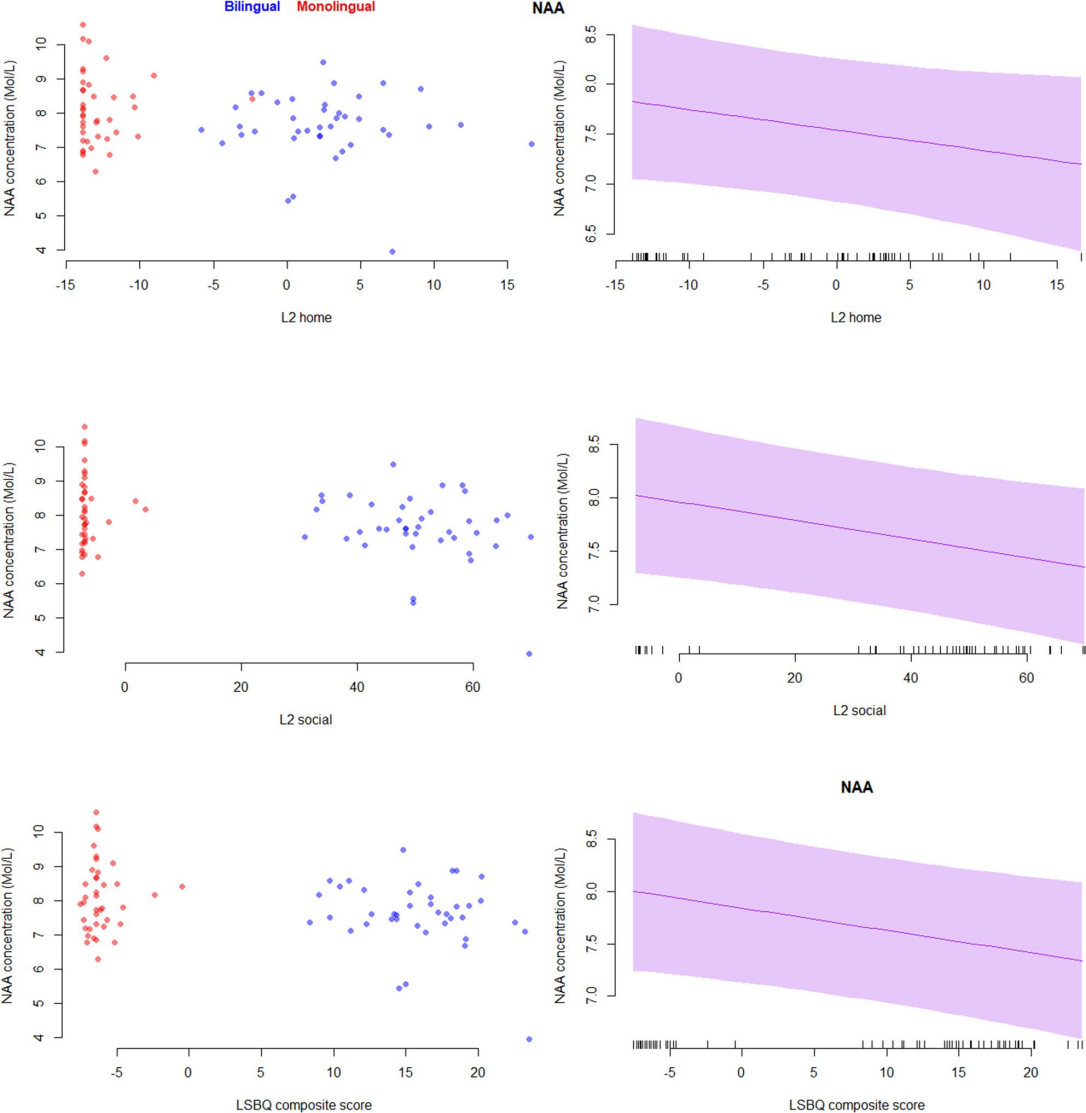
L2 home, L2 social and LSBQ composite scores as predictors of the absolute NAA concentrations. Shaded regions represent 95% confidence bands for the smoothed effects. Image produced in R ver. 4.0.3 (https://www.r-project.org/).

Our second set of analyses examined how metabolite concentrations relate to the degree of bilingual engagement across our entire group. This revealed that INS concentration was significantly positively correlated with all metrics of interest, L2 home, L2 social and the LSBQ composite score, in that the higher the experiences the higher the INS levels. Moreover, NAA concentration was significantly negatively correlated with the L2 social and the LSBQ composite scores, in that the higher the experiences the lower the NAA levels. The results from the second set of analyses are presented in Table 2. Figure 2 illustrates the effects on the INS concentrations, and Fig. 3 the effects on the NAA concentrations.

**Table 2:** The effects of L2 home, L2 social and LSBQ composite scores on the absolute metabolite concentrations, expressed in p values.

	INS	NAA	CHO	GLX	CRE
L2 home	0.035*	0.131	0.552	0.898	0.876
L2 social	0.025*^	0.023*^	0.412	0.690	0.690
LSBQ composite	0.028*^	0.033*^	0.534	0.752	0.686

For significant effects all Fs > 4.8

^edf > 1, denoting a non-linear effect.

## Conclusions

Building on findings that the cognitively challenging experience of bilingualism can have knock-on consequences for the structure and function of brain regions related to language acquisition and control, and the (functional and structural) connectivity between them^6,30,45^, the present study used MRS to investigate metabolic correlates of these adaptations. Recall that we focused on the concentrations of several well-understood metabolites in the basal ganglia, a region crucial for language selection and control in bilinguals^27^. Because the effects of bilingualism on the brain can vary as a function of engagement with relevant experience, our sample included a considerable group of individuals varying in degree of bilingual engagement factors. Given that age is a covariant factor, our sample also included a considerable range, enabling us to tease apart the effects of ageing from bilingualism. Our results revealed age effects that largely corroborate previous findings in this particular brain region^19^; specifically, INS concentration increased, CHO and CRE concentrations remained stable, and NAA and GLX concentrations decreased. Notably, the effects of age on NAA and GLX concentrations emerged as non-linear, a finding that might explain the inconsistencies in the literature, and which should be considered in future studies. Crucially, our results also revealed that our bilingual subgroup had higher INS and lower NAA concentrations than the monolingual one. Moreover, and in accordance with our prediction, our results showed that age was a significant predictor of CHO concentrations for the bilingual group only. Notably, when we examined the unified group accounting for age, the amount of bilingual experience was positively correlated with the INS concentration and negatively correlated with the NAA concentration, but did not significantly correlate to the CHO concentration.

We first turn to the effects on INS, interpreting them within the framework of bilingualism-induced neuroplasticity. INS is typically linked to increased activity of glial cells like astrocytes and microglia^46,47^. Increased levels of INS have been reported in the brains of blind individuals, interpreted to indicate repurposing of visual regions^21,22^, as well as for several diseases, including HD^10^, MCI/AD^11^ and MS^12^. In the cases of patient studies, increases are typically attributed to *astrogliosis,* a manifestation of abnormal proliferation of astrocytes due to the destruction of nearby brain cells following trauma or neurodegeneration. This interpretation explains our finding of increased INS levels with increased age: ageing brains suffer neuronal loss, a process which might be accompanied with or followed by astrogliosis^48^. However, this does not account for the higher INS concentrations observed in bilinguals, who had a comparable age-range to that of monolinguals. A distinct mechanism might be at play in this case.

Recall that elevated INS levels can also relate to increased microglia activation. Among other functions, microglia are thought to be involved in regulating synaptic activity, and notably in synaptic pruning^48^. This function might be key to interpreting the present findings. Recall that bilingualism, similar to other types of cognitively complex skill acquisition^4^, can induce temporal volumetric grey matter increases followed by renormalisation over increased experience of using the new skill^6^. In the case of bilingualism, this process particularly affects the basal ganglia, which are key to language control for bilinguals^27^, with the caudate nucleus and the putamen undergoing dynamic structural changes^6^. Crucially, this expansion-renormalisation process has been (at least tentatively) attributed to pruning of superfluous synapses that were formed during the skill acquisition, in order for the more efficient ones to be utilised^4^. Therefore, the increased INS concentration in the present study might be a marker of a bilingualism-specific process of expansion and renormalisation of regions related to controlling two languages. Since our bilingual participants were all residents of the UK at the time of testing, and thus active users of their L2 (English), they all had substantial experience in language switching and control, which should have contributed to optimisation of the language control system via processes like synaptic pruning. This interpretation is further supported by the finding that the INS concentration is positively correlated with the amount of bilingual experience; higher levels of bilingual experience led to more extensive renormalisations of the previously expanded structures of the basal ganglia, observed via higher INS concentrations as a function of more experience. Finally, this effect appears to be independent of age, as the interaction in our main analyses did not emerge significant. In other words, the significant effects of age in INS concentration are not affected by the language status of the individual (and vice versa); while both groups show the expected INS increases, bilinguals have elevated INS concentration across all ages. This suggests that the two effects (of age and of bilingualism) might be underpinned by different mechanisms. A related effect was observed in the concentrations of CHO, where age emerged as a significant predictor for the bilingual group only, while CHO concentrations remained unchanged in monolinguals, as predicted^17–19^. Similar to INS, increases in CHO concentrations have been linked to glial activity which underlies brain plasticity, usually in occipital regions^21,22^. Therefore, our pattern of results suggests that similar mechanisms may also apply to the basal ganglia, and potentially accelerated in older age in long-term bilinguals, such as the older participants in our sample.

The other significant finding pertinent to bilingualism relates to the concentration of NAA, a neurotransmitter primarily found in neurons^49^. The observed negative relationship with age was predicted and can be readily explained, given that NAA is considered a marker of neural density and its concentrations are expected to reduce with age, especially in the basal ganglia^19^. The observed lower concentration for bilinguals might appear counterintuitive if viewed from the same ageing perspective, as it would signify faster ageing for bilinguals; however, in the absence of an interaction between group and age, this interpretation cannot be supported. An alternative interpretation may be found in the particular properties of NAA, reductions of which have been linked to processes such as synaptic pruning in developmental studies^50^. As already mentioned, according to dynamic accounts of experience-based neuroplasticity, synaptic pruning is a key process underlying optimisation of neural networks, including, but not limited to, those underlying language control^4,6^. Viewed through this lens, our results suggest an optimised language control network for our bilinguals; at the same time, the significant correlations between the NAA concentration and our measures of bilingual experience suggest an ongoing, dynamic process. In this respect, our NAA findings are complementary to, and indeed mirror, those observed for INS: our bilinguals optimise their language control system via synaptic loss (decreased NAA levels) in the basal ganglia, which can be attributed to increased microglia activation (elevated INS levels), a process intensified with increased engagement with bilingual experiences.

The importance of these findings is, at least, twofold. First, the data shows that the well-documented effects of bilingualism on brain structure and function have their correlates in changes in brain metabolism. Crucially, we report markers of metabolic activity which are compatible with experience-based approaches, arguing for bilingualism-induced dynamic brain adaptations^6^. Given the important implications these adaptations may have for healthy and pathological ageing of the bilingual brain^51^, future studies should pay particular attention to these indices of neuroplasticity and how they interact with brain decline in key areas related to language processing and control. In doing so, future studies should also account for factors such as dietary habits, substance use and other environmental factors that might be critical in the case of older participants to get the best sense of what bilingual engagement contributes independently of other co-occurring factors. Second, and more generally, we show here that sustained, long-term cognitively challenging experiences, such as controlling two languages, might also have persisting effects on metabolite concentrations in the brain. Therefore, it is possible that similar long-term findings could be reported for other types of skill learning and experiences that have shown to restructure the brain (e.g., music, driving, exercise), and such effects are not limited to short-term training. Insofar as cognitively challenging experiences have a direct impact on metabolite concentrations in the healthy brain, they are useful in furthering our theoretical understanding of the mechanisms underlying skill acquisition and use, and the accompanying neural adaptations.

## Methods

### Participants

In total, 99 adults were recruited. Inclusion criteria for the study included being right-handed (self-reported), no history of speech and language disorders and no contraindication to MRI scanning. The participants were divided into two groups. The Bilingual group consisted of participants who spoke English as their second language (L2) and were resident in the UK at the time of testing, i.e., they were immersed in an L2-speaking environment. Importantly, there were no inclusion criteria relating to their native language or other language factors in order to recruit the widest possible range of linguistic experiences^37,38^. The English native comparison group (henceforth called “Monolingual” group for convenience) included individuals born and raised in the UK who had minimal or no exposure to additional languages, a typical and representative demographic in the UK. Of the participants that were recruited to the study, 20 were removed from the final cohort for the following reasons: two anticipated native speakers of English wound up being too highly exposed to a second language, therefore constituting outliers to their group; MRS spectrum could not be extracted for one participant due to a corrupted data file; four participants were outside the Cramér-Rao lower bound (CRLB) (3 for GLX and 1 for NAA) (see Data analysis for our choice of the CRLB threshold); for eleven participants visual inspection revealed that the manual voxel placement for the MRS scanning sequence was poor; finally, two participants in the bilingual group were excluded as their concentrations for four out of five metabolites of interest constituted extreme values, so we suspected undiagnosed underlying pathology. The final sample consisted of 79 participants (age range 19–83), including 39 monolinguals (25 female) and 40 bilinguals (29 female). See Table 3 for full descriptors of the final sample. This research was approved by the University of Reading Research Ethics Committee. Informed consent was obtained from all participants.

**Table 3:** Mean (SD) of group demographics and absolute metabolite concentrations.

	Bilingual	Monolingual
Age (years)	42.12 (15.64)	41.28 (21.45)
L2_Home	2.59 (4.51)	− 12.76 (2.11)
L2_Social	50.44 (9.89)	− 6.39 (2.30)
LSBQ composite	15.78 (3.85)	− 6.09 (1.31)
INS (Mol/L)	4.83 (1.00)	4.32 (1.03)
NAA (Mol/L)	7.60 (0.98)	8.10 (1.01)
CHO (Mol/L)	1.79 (0.22)	1.72 (0.26)
GLX (Mol/L)	12.45 (2.47)	11.83 (3.01)
CRE (Mol/L)	5.99 (0.80)	5.84 (1.21)

### Materials

Both participant groups completed the Language and Social Background Questionnaire (LSBQ)^52^ which documents the participants’ language use from childhood to the present day and across several settings and dimensions. The LSBQ yields two scores related to the amount of (bilingual) language use within specific communicative settings, which have been shown to predict bilingualism-induced changes in brain structure and function^37,38^. Specifically, L2 social corresponds to the degree of L2 exposure and use in societal and community settings whereas L2 home corresponds to the extent of L2 proficiency and use in home settings. Moreover, LSBQ outputs a composite score accounting for the overall bilingual experience. For all three scores, a higher value indicates increased bilingual engagement, i.e., increased (balance in) use of, and switching between, the two languages.

### MRI data acquisition

**Figure 4:**
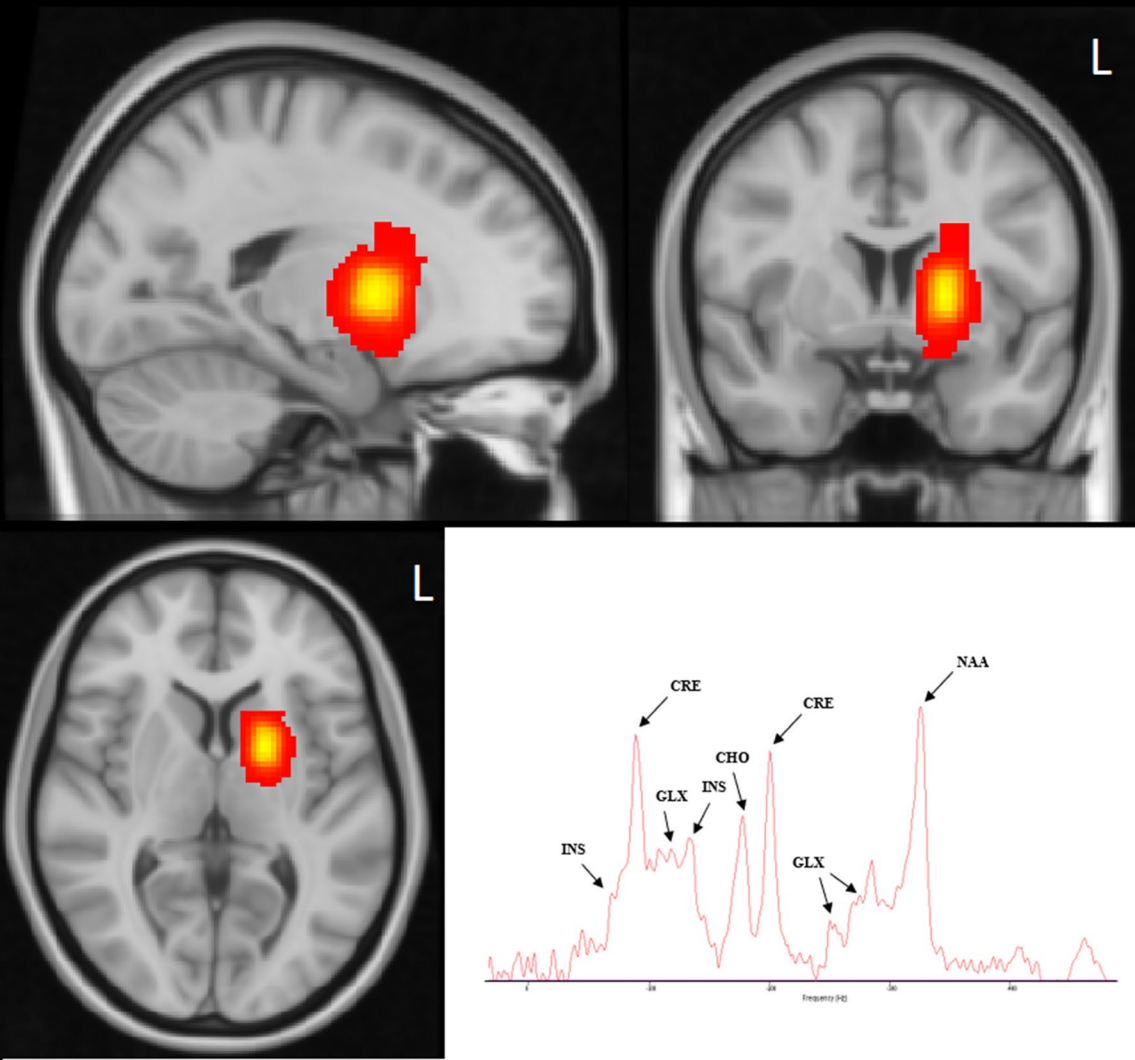
Location of the MRS voxels of the entire group in the left basal ganglia, shown in standard space. The heatmap was generated using Gannet3.0 (http://www.gabamrs.com/) and SPM12 (running in Matlab R2020b), and was visualised in FSLeyes 0.34.2 (FSL version: 6.0.4). Warmer colours represent greater voxel overlap between participants (peak coordinates: − 18, 4, 4). Bottom right presents a representative acquisition spectrum (produced with jMRUI ver. 5.2, http://www.jmrui.eu/).

Neuroimaging data were acquired on a 3 T Siemens MAGNETOM Prisma_fit MRI scanner, with a 32-channel Head Matrix coil and Syngo software. A high-resolution T1-weighted MPRAGE sequence was acquired (256 sagittal slices, voxel size: 0.7 mm isotropic, in-plane acquisition matrix: 320 × 320, echo time (TE) = 2.41 ms, repetition time (TR) = 2400 ms, inversion time = 1140 ms, flip angle = 8°). For purposes of voxel placement an T2-weighted (HASTE) scan was also run (15 coronal slices, voxel size 0.7 × 0.7 × 3mm, in-plane acquisition matrix: 320 × 320, TE = 82 ms, TR = 1500 ms, flip angle = 150°). Finally, a PRESS spin-echo sequence was used to acquire MRS data, involving a single voxel placed at the left basal ganglia (Fig. 4). (15 × 10 × 15mm, 128 averages, transversal orientation, TR = 2000 ms, TE = 30 ms, flip angle = 90°). Automatic shimming was used, and Chemical Shift Selective water Suppression (CHESS)^53^ was applied (bandwidth: 50 Hz).

### MRS data quantification

^1^H-MRS spectra were processed in the time domain within the software Java-Based Magnetic Resonance User Interface (jMRUI) software (version 5.2)^54^. Following literature in the field^18,44^ we focused on the concentrations of NAA, CHO, CRE, INS and GLX. In the preprocessing phase, the water spectrum acquired closest to the measurement was used to perform phase correction. Afterwards, a Gaussian filter of 3 Hz was applied on each spectrum to improve signal quality, decrease noise and reduce signal truncation effects^55^. Residual water peaks were removed with the Hankel-Lanczos Singular Value Decomposition (HLSVD) filter tool^56^. In the quantification phase, the same metabolite models as in^57^ were employed for NAA, CRE, INS and GLX; exceptionally, and following from recent literature^18,44^, CHO was modelled as a single peak derived from the sum of choline, phosphocholine and glycerophosphocholine peaks. Accurate Quantitation of Short Echo time domain signals (AQSES) was applied using the method described in^58^. The spectrum and the model were shifted and aligned so that the NAA peak was at 2.02 ppm in order to correct for any chemical shift displacement. Limitation of frequency range for processing was selected at 0–8.6 ppm. The Hankel Lanczos Total Least Squares (HLTLS) algorithm was applied (0 truncated points, 2048 points in AQSES and normalization turned on). Several constraints were imposed on the phases, dampings and frequencies within AQSES (equal phase for all metabolites, begin time fixed, delta damping (− 10 to 25 Hz), delta frequency (− 5 to 5 Hz), no background handling). To account for differences in grey matter volume (GMV) in the voxel, partial volume correction was performed according to the procedure described in^59^: specifically, metabolite values were corrected to account for differences in gray matter (GM), white matter (WM) and cerebrospinal fluid (CSF) within the voxel. Moreover, T1 and T2 values from GM and WM at 3 T were used as base to compute attenuation factors for both water and metabolites^60–62^. These factors were in turn used to correct the reported values for relaxation effects dependent on voxel’s tissue proportion. Table 3 illustrates the mean absolute concentrations for our five metabolites of interest.

### Data analysis

Rejection criteria included one or more of the participants metabolite concentrations being outside the Cramér–Rao lower bound (CRLB)^63^. Setting the appropriate CRLB threshold remains controversial in the relevant literature^64^, and thresholds can vary from 20 to 50%, depending on the metabolites and regions of interest^57,65,66^. Following from research looking at the same brain region^67,68^, we chose a CRLB threshold of 30% which gave us good data quality.

The corrected and quantified MRS data were analysed in R^69^ with generalised additive models (GAMs), by using the bam() function of the mcgv package^70^. Since our sample covers a large age range (19–83 years), and the potential effects of age on metabolite concentrations are not well understood, GAMs were selected as a method that can account for potential non-linear effects of age on brain measures^71–73^. Specifically, GAMs fit a nonlinear regression spline consisting of the sum of simpler nonlinear functions, but this is only included where there is sufficient evidence for a particular curve. GAMs report the nonlinearity of the effect in the form of estimated degrees of freedom (edf), where edf = 1 denotes a linear term and edf > 1 a nonlinear term. Separate GAMs were run for each metabolite of interest and in two sets of analyses (all tests two-sided) (see Supplemental Material 1 for the full analysis code).

The first set of analyses looked at the effects of age on metabolite concentrations, and how these might interact with bilingualism. In a first-level model (Model 1) we applied a GAM in which we fit a regression spline for the effects of Age, along with random effects of Participant and Sex. In the second-level model (Model 2) we included a term for Bilingualism as an ordered factor (which allows estimating interactions), a regression spline term for Age, a smoothed term for the Age x Bilingualism interaction, as well as the random effects of Participant and Sex. This model was run twice, once for each level of Bilingualism as the reference level, following an analytical procedure akin to using a “vibration of effects” approach^73,74^. In cases were the Age x Bilingualism interaction emerged reliably significant (i.e., in both versions of Model 2), a third- level model (Model 3) was run, in order to unpack the interaction. This model included a main effect of Bilingualism and smooth terms for Age for each level of Bilingualism, along with random effects of Participant and Sex.

The second set of analyses looked at the effects of language experiences on the concentrations of the metabolites of interest across the entire group. These analyses were run separately for each of the three metrics of the LSBQ, namely L2 Home, L2 Social and Composite scores. Similarly to the first set of analyses, in this model (Model 4) we applied a GAM in which we fit a regression spline for the effect of the metric, a regression spline for the effect of Age, along with random effects of Participant and Sex. The entire sample of participants was included in this analysis without splitting them by their bilingual status. This was because we collected LSBQ scores from our native speakers too, and several of them reported some limited experience with other languages.

### Ethical approval

This research was performed in accordance with the Declaration of Helsinki. It was approved by the University of Reading Research Ethics Committee. Informed consent was obtained from all participants.

## Supplementary Information

Supplementary Information.

## Data Availability

The data analysed for this study are included in this published article as a supplementary information file.

## Code availability

The code used for data analysis in this study is included in this published article as a supplementary information file.
