# Supplementary material for: Bilingualism is a long-term cognitively challenging experience that modulates metabolite concentrations in the healthy brain
Source: Sci Rep. 2021 Mar 29;11:7090. doi: 10.1038/s41598-021-86443-4 (PMC8007713; doi:10.1038/s41598-021-86443-4)
Supplement: Supplementary file 1 — Supplementary Information. [file 41598_2021_86443_MOESM1_ESM.pdf]

Supplementary materials for:

Bilingualism is a long-term cognitively challenging experience that modulates metabolite concentrations in the healthy brain

Pliatsikas, C. (ORCID: 0000-0001-7093-1773)[a,b*], Pereira Soares, S.M. (ORCID: 0000-0002-1709-6059)[c] , Voits, T. (ORCID: 0000-0001-7223-8446)[d], Deluca, V. (ORCID: 0000-0002-2275-209X)[d], & Rothman, J. [b,d*]

[a]School of Psychology and Clinical Language Sciences, University of Reading, Harry Pitt Building, Earley Gate, Whiteknights Road, Reading, RG6 6AL, UK

[b] Centro de Ciencia Cognitiva, Facultad de Lenguas y Educación, Universidad Antonio de Nebrija, Calle de Sta. Cruz de Marcenado, 27, 28015, Madrid, Spain

[c]Department of Linguistics, University of Konstanz, Universitätsstraße 10, 78464 Konstanz, Germany

[d]Department of Language and Culture, The University of Tromsø, Hansine Hansens veg 18, 9019, Tromsø, Norway

Contents:

1. Code for the MRS analysis
2. Data for the MRS analysis

1. Code for the MRS analysis (INS used as an example, the same models were applied to all metabolites).

A) First sets of analyses: The effects of age on metabolite concentrations and how it interacts with bilingualism

a) Model 1:First GAM looking at the smoothed effect of age. Subject and sex treated as random effects

```
----------
m.age <- bam(INS ~ s(subject, bs="re") + s(sex, bs="re") + s(Age), data=dat.placement, method="REML")
----------
```

b) Model 2: Second GAM with bilingualism (marked as “nativeness”) as ordered factor, looking at age X bilingualism interactions, examined for each level of bilingualism separately. This will only produce p values for the interaction, and will need to be followed up with Model 3 for the interaction to be unpacked. Subject and sex treated as random effects.

```
----------
# Ordered factor for nativeness, and set contrasts. Ref= Native

dat.placement$nativeness <- relevel(dat.placement$nativeness, ref="Monolingual")
dat.placement$OFnativeness <- as.ordered(as.factor(dat.placement$nativeness))
contrasts(dat.placement$OFnativeness) <- 'contr.treatment'
contrasts(dat.placement$OFnativeness)


m.native <- bam(INS ~ s(subject, bs="re") + OFnativeness  + s(Age) + s(sex, bs="re") + s(Age, by=OFnativeness),
        data=dat.placement, method="REML")

######################################################################

# Ordered factor for nativeness, and set contrasts. Ref= Non-native

dat.placement$nativeness <- relevel(dat.placement$nativeness, ref="Bilingual")
dat.placement$OFnativeness <- as.ordered(as.factor(dat.placement$nativeness))
contrasts(dat.placement$OFnativeness) <- 'contr.treatment'
contrasts(dat.placement$OFnativeness)

m.nonnative <- bam(INS ~ s(subject, bs="re") + OFnativeness  + s(Age) + s(sex, bs="re") + s(Age,
by=OFnativeness), data=dat.placement, method="REML")
----------
```

c) Model 3: Third GAM, looking at each level of Nativeness separately and producing separate smooths per group. Only useful if the agexnativeness interaction is significant above. Subject and sex treated as random effects

```
----------
m.all <- bam(INS ~ s(subject, bs="re") + nativeness + s(sex, bs="re") + s(Age, by=nativeness), data=dat.placement, method="REML") #+ s(sex, bs="re")

---------
```

B) Second sets of analyses: The effects of bilingual experiences on metabolite concentrations.

L2 home

```
----------
m.home <- bam(INS ~ s(subject, bs="re") + s(sex, bs="re") + s(Age) + s(L2_home), data=dat.placement, method="REML")
----------
```

L2 social

```
----------
m.social <- bam(INS ~ s(subject, bs="re") + s(sex, bs="re") + s(Age) + s(L2_social), data=dat.placement, method="REML")
----------
```

LSBQ composite score

```
----------
m.LSBQ_composite <- bam(INS ~ s(subject, bs="re") + s(sex, bs="re") + s(Age) + s(LSBQ_composite), data=dat.placement, method="REML")
----------
```

2. Data for the MRS analysis

subj_ID	Age	sex	nativeness	L2_home	L2_social	LSBQ_composite	NAA	CHO	CRE	INS	GLX
207	34	male	Bilingual	3.550585	65.7929	20.18914	8.008563	1.639157	5.006795	5.581107	14.13168
209	29	female	Bilingual	-3.19523	48.27492	12.63316	7.603525	1.954797	6.93068	5.188147	13.14111
218	26	female	Bilingual	-3.49727	32.91367	8.975423	8.167665	1.456001	5.911479	5.125188	10.4014
224	22	female	Bilingual	3.291654	59.55359	19.0498	6.693237	1.598078	5.84457	4.752427	12.34596
241	38	female	Bilingual	0.764027	48.30836	14.01929	7.459018	1.797483	5.928784	4.259169	11.88284
243	43	female	Bilingual	3.170246	57.99857	18.46426	8.871973	1.782258	6.683784	2.740189	13.62335
244	25	male	Bilingual	4.342705	49.38355	16.34564	7.085086	1.814636	6.006274	3.870075	13.09012
248	29	female	Bilingual	4.876748	59.25342	18.50657	7.830807	1.604681	6.035745	4.39152	12.38752
249	23	male	Bilingual	-3.11575	30.88868	8.335885	7.357085	1.810906	4.743173	4.647005	12.40481
250	39	male	Bilingual	1.385641	60.5744	18.06709	7.486995	1.977397	5.610774	4.816179	11.33282
251	29	female	Bilingual	-2.43356	38.65394	11.04853	8.577391	1.791441	4.918966	3.483437	11.47176
252	32	male	Bilingual	6.946029	69.80065	22.51195	7.3696	1.487552	4.978321	4.298583	14.02637
253	29	male	Bilingual	-5.80879	40.39611	9.71842	7.5142	1.991299	6.682214	3.4418	15.16483
254	31	female	Bilingual	0.400863	63.98911	19.34753	7.859622	1.833117	5.984387	3.757442	12.13439
255	24	female	Bilingual	2.530505	52.63166	16.72204	8.09779	1.606077	6.325123	5.519588	11.43394
256	22	female	Bilingual	-1.75885	33.786	9.73183	8.582506	1.755706	5.32363	3.530695	11.77344
257	38	female	Bilingual	2.249035	45.01869	14.34527	7.572985	1.603185	5.66613	4.87725	16.27221
259	35	female	Bilingual	2.223283	38.02985	12.24012	7.316008	1.537217	5.016367	4.339403	15.91006
260	30	female	Bilingual	4.87878	49.03132	15.81667	8.494158	1.606888	5.753316	4.906855	9.907286
266	39	female	Bilingual	0.451083	54.2632	15.78636	7.277942	1.514412	5.939541	4.097631	14.95244
268	46	female	Bilingual	3.779764	59.24558	19.11848	6.884367	1.947019	5.615147	5.360995	14.46953
269	26	female	Bilingual	-4.42724	41.18843	11.15096	7.113677	1.925214	6.93917	4.741477	13.97065
301	30	male	Monolingual	-11.6104	-6.99641	-5.67492	7.442661	1.734566	5.583339	5.165124	13.37796
302	23	female	Monolingual	-13.8999	-6.99641	-6.43046	9.299258	1.627601	6.283206	4.372204	10.52379
308	30	male	Monolingual	-13.8999	-6.99641	-7.16939	7.198481	1.66014	5.199316	2.806057	12.30135
310	24	female	Monolingual	-13.8999	-6.99641	-6.43046	8.247853	1.634536	7.158103	3.743598	14.32059
311	25	female	Monolingual	-9.04948	-6.99641	-5.27827	9.095084	1.561434	7.195243	3.702793	13.32561

313	27	male	Monolingual	-13.8999	-6.99641	-6.43046	7.726582	1.960256	5.658863	3.990563	13.44759
314	26	male	Monolingual	-13.8999	-6.99641	-6.43046	9.20207	1.798793	5.893945	2.780236	10.46069
316	21	female	Monolingual	-13.4959	-6.99641	-6.29715	8.830567	1.635448	5.522427	2.897878	13.1361
320	19	female	Monolingual	-12.0807	-4.77644	-5.16415	6.769927	1.591871	5.24363	4.738962	13.84983
321	25	female	Monolingual	-13.8999	-6.99641	-6.43046	8.136157	1.841254	5.963653	4.639456	13.23349
322	21	female	Monolingual	-13.8999	-6.99641	-6.43046	8.672343	1.617174	4.791755	4.320638	11.40041
324	20	female	Monolingual	-13.8999	-6.99641	-6.43046	6.859675	1.907638	5.935883	5.801581	14.33464
325	27	female	Monolingual	-13.8999	-6.99641	-7.16939	8.104608	1.738941	5.607038	4.033263	11.71325
326	23	female	Monolingual	-12.2533	-6.99641	-5.88708	7.240777	1.425703	5.92834	3.123237	12.03564
328	19	female	Monolingual	-13.4959	-6.99641	-6.29715	10.0837	1.861206	6.666475	3.343995	15.18576
329	23	female	Monolingual	-12.9592	-6.99641	-6.12003	7.71888	1.558116	6.750302	3.901528	15.15684
331	22	female	Monolingual	-13.8999	-6.99641	-6.43046	7.617131	1.68993	5.646115	4.171691	14.12534
332	21	male	Monolingual	-13.8999	-6.99641	-7.52943	7.907956	2.163476	7.271145	4.795062	15.04319
334	24	female	Monolingual	-13.8999	-6.99641	-6.43046	10.17399	1.330331	5.683704	3.209748	8.400046
201	55	male	Monolingual	-10.3619	3.505831	-2.38455	8.165671	1.652915	5.890431	4.279531	13.53752
202	59	male	Monolingual	-13.8999	-7.49993	-7.06182	6.772788	1.652178	5.477714	5.30828	10.23518
203	61	female	Monolingual	-11.8062	-7.49993	-5.89061	8.469945	2.060677	5.881005	4.883331	10.9368
207	70	female	Monolingual	-13.3355	-7.49993	-6.98641	6.979841	1.781191	5.800453	5.434997	8.709021
213	83	male	Monolingual	-10.5088	-5.84085	-4.96474	8.478625	1.967056	7.333049	4.559459	9.176484
223	68	female	Monolingual	-10.1567	-5.52383	-4.75343	7.320871	1.652505	4.357769	5.405335	9.040485
224	71	male	Monolingual	-13.1396	-7.49993	-7.16334	8.476823	1.932696	6.607141	5.845966	12.87584
225	60	male	Monolingual	-13.0303	-7.49993	-6.29455	6.292473	1.824423	7.215085	4.551777	15.23279
226	73	male	Monolingual	-12.9118	-6.68541	-6.0111	7.767585	2.067138	7.51993	4.97572	14.44702
230	75	male	Monolingual	-13.8999	-7.49993	-7.28464	7.445232	1.305691	7.313136	5.590899	9.834626
233	57	male	Monolingual	-13.8999	-7.49993	-7.27791	7.943071	1.882559	7.246493	6.049058	13.1687
235	48	female	Monolingual	-12.0811	-2.83625	-4.58223	7.812516	1.781655	5.229913	4.008034	11.08841
237	56	female	Monolingual	-13.8999	-7.49993	-6.58151	6.895801	1.844363	6.453504	3.425084	12.492
238	49	female	Monolingual	-13.8999	-7.49993	-6.7293	8.907114	1.437258	5.033313	4.346947	8.673062
101	53	female	Bilingual	0.351805	33.96132	10.40197	8.402285	2.073296	7.716453	4.787243	15.01307
102	51	female	Bilingual	6.547357	55.72576	18.89686	7.498968	1.678735	5.047073	4.4269	9.373196

103	64	female	Bilingual	2.563382	47.65862	15.24098	8.243123	1.991031	7.138425	7.008239	10.54586
105	57	female	Bilingual	6.539027	54.6679	18.21853	8.874325	1.677824	5.833972	6.396663	7.914723
106	52	female	Bilingual	2.236408	56.57956	17.69316	7.339272	2.157255	6.255431	5.195489	11.78413
108	56	female	Bilingual	9.061542	58.50445	20.19576	8.699361	1.884319	6.051091	5.046823	13.39867
109	71	female	Bilingual	3.910219	51.06628	16.70773	7.897941	1.721613	5.990857	5.743781	6.625144
112	73	female	Bilingual	3.354946	47.14839	15.27016	7.841561	2.042209	5.772915	5.082066	12.5078
114	50	male	Bilingual	0.417173	49.60006	14.96738	5.553527	1.972817	5.864679	6.075691	16.76257
115	52	female	Bilingual	11.79508	50.48155	17.22732	7.646674	1.773152	5.73445	4.972119	11.88425
116	57	female	Bilingual	9.649354	48.34441	17.78509	7.597849	2.125372	6.376285	4.52734	14.08079
118	58	male	Bilingual	16.61132	63.81622	23.23435	7.095177	1.721508	7.315759	6.492977	11.05229
123	70	female	Bilingual	2.954011	43.71407	14.18652	7.619049	2.179433	5.792213	5.246332	11.31034
125	60	male	Bilingual	0.081886	49.59671	14.48768	5.443181	1.251579	4.814714	6.069068	8.757588
126	63	female	Bilingual	-0.64692	42.36119	12.0751	8.303105	2.224168	7.106475	5.554899	14.53383
215	73	female	Monolingual	-2.37388	1.728167	-0.49489	8.406047	1.427774	4.159316	4.467166	4.815673
232	67	female	Monolingual	-12.882	-6.84776	-6.41944	7.32096	1.19153	3.103675	2.559465	5.238296
234	66	female	Monolingual	-13.5941	-7.49993	-6.88703	7.16397	1.183107	3.350828	2.21937	10.16043
107	58	male	Bilingual	7.184399	69.53869	23.48788	3.96589	1.614792	4.957261	3.992421	13.12944
210	22	female	Bilingual	2.426765	46.16367	14.82639	9.479559	1.652671	5.889004	2.477294	6.774836
261	29	male	Bilingual	-2.20572	49.98278	14.36442	7.470586	1.764684	8.102814	6.533142	16.61649
306	23	female	Monolingual	-13.8999	-6.99641	-6.43046	8.657418	2.375065	8.197945	6.139225	18.99256
319	21	male	Monolingual	-12.3163	-6.99641	-6.61963	9.610962	1.610741	3.195396	3.225318	4.760181
333	25	female	Monolingual	-13.8999	-6.99641	-6.43046	10.58399	2.1138	4.547246	5.689726	12.48337
